# HDGNN-Mamba2: Mamba-Based Spatiotemporal Heterogeneous Dynamic Graph Neural Network for Major Depressive Disorder Classification

**DOI:** 10.3390/brainsci16080872

**Published:** 2026-08-17

**Authors:** Jian Yan, Renzhou Gui, Hao Liang, Yaqi Wang

**Affiliations:** College of Electronic and Information Engineering, Tongji University, Shanghai 201804, China2111102@tongji.edu.cn (H.L.);

**Keywords:** major depressive disorder, functional magnetic resonance imaging, structured state space duality, graph neural network, deep learning

## Abstract

**Highlights:**

**What are the main findings?**

- HDGNN-Mamba2, a novel Mamba2-GNN hybrid with cross-attention fusion, jointly models temporal dynamics and spatial topology of fMRI brain networks, overcoming the limitations of Transformers and existing GNNs.
- The framework achieves 83.88% accuracy on REST-meta-MDD, demonstrating competitive performance among GNN-based approaches, with higher sensitivity (86.52%).

**What are the implications of the main findings?**

- The heterogeneous graph module integrates spatiotemporal brain features with non-imaging demographic data via dynamic edge updating, enabling more comprehensive subject representations beyond existing approaches.
- The framework identifies interpretable MDD-related model-attributed regions consistent with existing studies, demonstrating its potential as an exploratory framework for MDD classification using multimodal brain imaging and demographic data.

**Abstract:**

**Background**: Major depressive disorder (MDD) affects 332 million people worldwide, yet diagnosis remains reliant on subjective clinical interviews with substantial inter-rater variability. Objective neuroimaging model-attributed regions offer a path toward precision psychiatry, but existing computational approaches often lack clinical interpretability. **Methods**: We propose HDGNN-Mamba2, a Mamba-based spatiotemporal heterogeneous dynamic graph neural network. A hybrid Mamba2-GNN block with cross-attention fusion is developed to capture individual spatiotemporal contextual features and identify model-attributed regions. A heterogeneous global graph block with dynamic edge updating is constructed, integrating individual brain features with non-imaging phenotypic information (sex, age, education) to extract embeddings through inter-individual relationship modeling. Heterogeneous Graph Supervised Contrastive Learning is integrated to enhance discriminative capacity. **Results**: Evaluated on 533 subjects from the REST-meta-MDD dataset, HDGNN-Mamba2 achieved 83.88% accuracy, 86.52% sensitivity, and 80.85% specificity in ten-fold cross-validation. The identified model-attributed regions include the anterior cingulate cortex, parahippocampal gyrus, and thalamus. **Conclusions**: HDGNN-Mamba2 demonstrates competitive performance as an algorithmic framework for MDD classification, offering complementary architectural advantages in spatiotemporal fusion and interpretable region identification.

## 1. Introduction

Major depressive disorder (MDD) is a common mental disorder that places a substantial burden on affected individuals [1]. According to the latest World Health Organization estimate, approximately 332 million people worldwide have depression [2]. Depression is characterized by persistent low mood and loss of interest or pleasure. It can cause psychological and physical problems that affect work, study, appetite, sleep, and the ability to enjoy daily activities [3]. MDD may also involve suicidal thoughts or behaviors [4]. MDD is diagnosed mainly through clinical interviews and questionnaires based on self-reported symptoms. These procedures can be time-consuming and depend partly on clinical judgment, which may make early diagnosis difficult. MDD is also heterogeneous in clinical presentation, prognosis, and treatment response [5]. Objective and efficient methods are therefore needed to support MDD classification and identify model-attributed brain regions [6]. Recent advances in artificial intelligence and machine learning have shown promise in biomedical research, including disease diagnosis and drug discovery [7].

Currently, various medical imaging techniques are available to enhance the diagnosis and understanding of neurological disorders, such as positron emission tomography (PET), electroencephalography (EEG), functional magnetic resonance imaging (fMRI), magnetoencephalography (MEG), and diffusion tensor imaging (DTI) [8]. Among those medical imaging methods, fMRI possesses characteristics such as non-invasiveness, high spatial resolution, and multidirectional imaging capabilities for detecting brain activity, and has been widely used to investigate the pathogenesis of MDD [9]. Liang et al. discovered the neural subtypes related to MDD in resting-state functional magnetic resonance imaging (rs-fMRI) data, which may provide valuable evidence for understanding the heterogeneity of depression [10]. These findings indicate that fMRI can deepen our understanding of MDD pathophysiology and may inform future diagnostic approaches. Through the fusion of multimodal data, including fMRI data, psychosocial cognition, and social information, deep learning (DL) and machine learning methods are applied to the intervention, diagnosis, and management of psychiatric disorders, particularly depression [11].

In recent studies, the machine learning algorithms most frequently employed for classifying depression include K-Nearest Neighbor (KNN), Logistic Regression (LR), Support Vector Machine (SVM), Decision Tree, and Naive Bayes (NB) [12]. Yamashita et al. developed the logistic regression algorithm to distinguish participants with MDD from healthy controls, achieving a classification accuracy of approximately 70% [13]. Using the Extreme Gradient Boosting (XGBoost) method, Shi et al. developed an rs-FC model that outperformed other approaches in classifying participants with MDD versus healthy controls at the individual level, reaching an accuracy of 72.8% [14]. However, traditional machine learning approaches do not support end-to-end learning and have significant limitations, as classification results largely depend on the importance of features obtained in the initial phase, while also exhibiting deficiencies in capturing topological and temporal features.

The ability of DL methods to perform automatic feature extraction and establish end-to-end classification models has driven their increasing application in the field of mental health diagnosis. Gupta et al. adopted a DNN (deep neural network) framework combined with hierarchical node pruning. This technique not only minimized the initial parameter count but also yielded 76.4% accuracy for MDD classification, while simultaneously mitigating the risk of overfitting [15]. Wei et al. developed a multi-stream 3D CNN architecture that allowed for the simultaneous training of features from sMRI and rs-fMRI modalities. The model achieved a 69.38% accuracy rate in classifying participants with MDD versus healthy controls [16]. Using a Transformer-Encoder architecture, Dai et al. extracted functional connectivity features from large-scale multi-site rs-fMRI data to differentiate between participants with MDD and healthy controls, achieving a mean classification accuracy of 78.11% [17]. The complex topological nature of brain networks poses a fundamental challenge for conventional deep learning models like CNNs, which are not inherently designed to accommodate non-Euclidean data. Graph convolutional neural networks present a promising approach for processing fMRI data, as such data inherently reflect the functional architecture of the human brain [18]. Venkatapathy et al. proposed a graph neural network-based ensemble model capable of distinguishing participants with MDD from healthy controls and, additionally, first-episode from recurrent depression. Tested on a large fMRI dataset comprising 821 participants with MDD and 765 healthy controls, the model attained 71.18% accuracy with upsampling and 70.24% accuracy with downsampling in the MDD versus HC classification [19]. Dai et al. introduced a dynamic feature extraction approach that computed time-point-specific Pearson correlations between brain regions to capture functional connectivity, which was then input into a graph convolutional network (GNN) for MDD classification, attaining 75.8% accuracy and revealing MDD-related brain regions [20].

Demographic information has been consistently recognized in clinical research as an important factor in the diagnosis of neurological disorders. Xing et al. achieved 90.0% accuracy on the ADNI II dataset by using demographic features as auxiliary outputs to classify normal controls and Alzheimer’s disease patients [21]. Liu et al. introduced a Multi-channel Fusion GNN that utilizes dual channels to extract features from low-order and high-order brain graphs. By incorporating demographic data via one-hot encoding, the model effectively identified MDD-associated brain regions, achieving 77.6% accuracy [22]. Building on insights from prior studies, this paper incorporates demographic data as supplementary input features.

Emotional instability over time is a common feature in psychiatric patients, which raises the possibility that their brain network connectivity patterns are not fixed but instead evolve dynamically. The fMRI signals are time series data, and the aforementioned deep learning methods primarily extract features from the brain’s spatial domain. This focus may limit their ability to capture temporal dynamics of inter-regional interactions. Recurrent Neural Networks (RNNs) are suitable for time series feature extraction, but suffer from long-term dependency issues, leading to information loss [23]. While Transformer-based models demonstrate superior performance in capturing global dependencies, the computational complexity of self-attention grows quadratically with image size, resulting in significant overhead [24]. Recently, modern structured state space sequence models (SSMs) such as Mamba have demonstrated strong performance in long-sequence modeling [25,26], achieving linear scaling with input length while efficiently representing dependencies at both global and local levels [27,28,29].

This paper proposes HDGNN-Mamba2, a Mamba-based spatiotemporal Heterogeneous Dynamic Graph Neural Network, for the classification of participants with MDD and healthy controls. In light of the importance of capturing spatiotemporal patterns from fMRI, the network constructs a heterogeneous graph network by fusing multimodal data such as spatiotemporal features extracted from individual subject fMRI time series and demographic phenotypic information, attaining competitive predictive accuracy via an end-to-end framework. Moreover, the proposed method identifies significant model-attributed regions for disease classification. First, a hybrid Mamba2-GNN block with a cross-attention Fusion mechanism is designed to combine Mamba with graph convolutional networks for extracting individual spatiotemporal contextual features from the fMRI data and to identify model-attributed regions. In this block, the Conv-SSD layer is used as the temporal layer to capture temporal dependencies within fMRI signals. The Conv-SSD layer utilizes a selective state space mechanism to perform encoding and representation learning on the input sequences, enabling it to capture dependencies across different positions in the sequence. Once the time series of each brain region has been processed by the temporal layer, the outputs are passed to the spatial layer, where a graph attention network is used to model spatial correlations between brain regions. Functional connectivity is quantified by Pearson correlation between regional fMRI time series. Furthermore, the graph network incorporates a self-attention-based pooling module, where a multi-head attention mechanism adjusts node scores to retain important nodes while eliminating redundant ones. These preserved high-importance nodes contain model-attributed regions relevant to MDD prediction. Second, a heterogeneous global graph is constructed to capture subject-level relationships by leveraging embeddings derived from individual brain features and non-imaging demographic data. The spatiotemporal features of individual brain regions are treated as node information in the heterogeneous graph. A clustering method is used to compute distinct inter-subject relationships from each phenotypic dataset, which are then encoded as different types of edges. The self-attention mechanism was employed to fuse information across edge types, obtaining heterogeneous global graph network feature embeddings that integrate diverse characteristic types. Finally, the HDGNN-Mamba2 model enhances the classification performance of MDD by capturing the most essential embedded features through the integration of multimodal data, including individual brain network characteristics and global inter-subject correlation features.

The contributions of this paper can be summarized as follows:

1. We proposed a novel spatiotemporal heterogeneous dynamic graph neural network framework for classifying MDD using fMRI data. This model extracts spatiotemporal feature embeddings from individual subject brain data and identifies model-attributed regions, while enhancing semantic information through heterogeneous network fusion of multimodal data including individual spatiotemporal brain features and diverse non-imaging phenotypic information between subjects.
2. A cross-attention Fusion mechanism is designed that enables bidirectional interaction between temporal and spatial features in the Mamba2-GNN block, where temporal dynamics and spatial topologies mutually enhance each other through cross-modal attention, capturing complex spatiotemporal dependencies that cannot be represented by independent processing.
3. A dynamic edge updating mechanism is proposed for heterogeneous graph neural networks, which periodically refines inter-subject connectivity based on evolving node embeddings. A learnable gating fusion strategy balances static phenotypic priors with dynamic feature similarities, enabling the graph topology to co-adapt with the representation learning process.
4. A Heterogeneous Graph Supervised Contrastive Learning module is integrated into the heterogeneous graph training, which explicitly promotes within-class cohesion and between-class separation in the embedding space by pulling embeddings of same-class subjects closer and pushing embeddings of different-class subjects apart, thereby providing complementary supervisory information to the classification loss.
5. An attention-based pooling approach is introduced to boost interpretability in the identification of key model-attributed regions, as well as to select the most discriminative spatiotemporal feature embeddings derived from brain regions of individual subjects.

## 2. Preprocessing and Data

### 2.1. fMRI Dataset

This study employs data derived from the REST-meta-MDD dataset. To our knowledge, the research team led by Yan Chaogan at the Institute of Psychology, Chinese Academy of Sciences, collaborated with twenty-five depression research teams spanning 17 institutions across China, collecting and processing MDD data following standardized protocols, ultimately developing the REST-meta-MDD dataset [30]. The REST-meta-MDD dataset has effectively integrated data from 1128 healthy control subjects and 1300 depression patients across various research centers. In particular, it is currently the world’s largest depression-related brain functional imaging database, containing participants’ demographic information, and has been made openly accessible to researchers worldwide. Specifics regarding data collection details may be accessed via the Supplementary Table on the website (http://rfmri.org/REST-meta-MDD (accessed on 12 August 2026)). In this study, we focused on the S20 cohort. The S20 cohort includes 533 subjects, comprising 282 participants with confirmed MDD diagnoses and 251 healthy individuals. Beyond the BOLD fMRI time series data, sociodemographic factors for each participant, such as age, sex, and duration of schooling, sourced from the REST-meta-MDD project, are incorporated in this study.

The demographic characteristics of the S20 cohort are summarized in Table 1. The MDD and healthy controls (HC) groups did not differ significantly in age or sex distribution. However, the HC group had significantly more years of education than the MDD group. This disparity is consistent with previous reports that depression is associated with lower educational attainment in clinical samples.

### 2.2. Data Preprocessing

The DPARSF toolkit [31] (http://www.rfmri.org/DPARSF (accessed on 12 August 2026)) was employed for data preprocessing. The preprocessing workflow included the following steps: (1) The initial ten volume features of the rs-fMRI data were excluded to achieve stability in the scanner’s magnetic field gradient. (2) Head motion correction was performed to realign images using a six-parameter rigid-body linear transformation. (3) T1-weighted structural scans were aligned with the mean functional volume via a linear transformation with six free parameters and no resampling. (4) T1-weighted images underwent segmentation into three tissue types: white matter, gray matter, and cerebrospinal fluid. (5) All acquired imaging datasets underwent normalization to align with the MNI standard anatomical space. (6) Head movement confounds were eliminated via regression based on the 24-parameter Friston model. (7) Bandpass filtering was applied to all time series within 0.01–0.1 Hz.

### 2.3. Functional Connectivity

The construction of brain functional connectivity mainly involves three steps: the parcellation of brain regions of interest, the extraction of time series, and the calculation of the indexes of brain functional connectivity.

(1) Regions of Interest

Following the preprocessing of fMRI data, it is essential to segment the regions of interest within the three-dimensional brain image. The most common parcellation approach uses brain atlases, which map voxels to anatomical or functional regions. Regional feature values are then computed by voxel-wise averaging or clustering. The segmentation of brain regions further transforms the large voxel point feature into a more macroscopic brain region-level feature, which is helpful for further analysis and more interpretable. Common brain atlases include: AAL (Automated Anatomical Labeling), Craddock, Harvard Oxford (HO), and POWER, among others. The POWER atlas was selected in this paper. Anatomical atlases such as AAL exhibit relatively poor within-ROI functional homogeneity and may not accurately reproduce voxel-level functional connectivity patterns, whereas functional parcellations provide superior homogeneity in this regard [32]. The cerebral imaging data are parcellated into 264 functional regions of interest based on the POWER functional parcellation template.

(2) Extraction of Time Series

**Figure 1:**
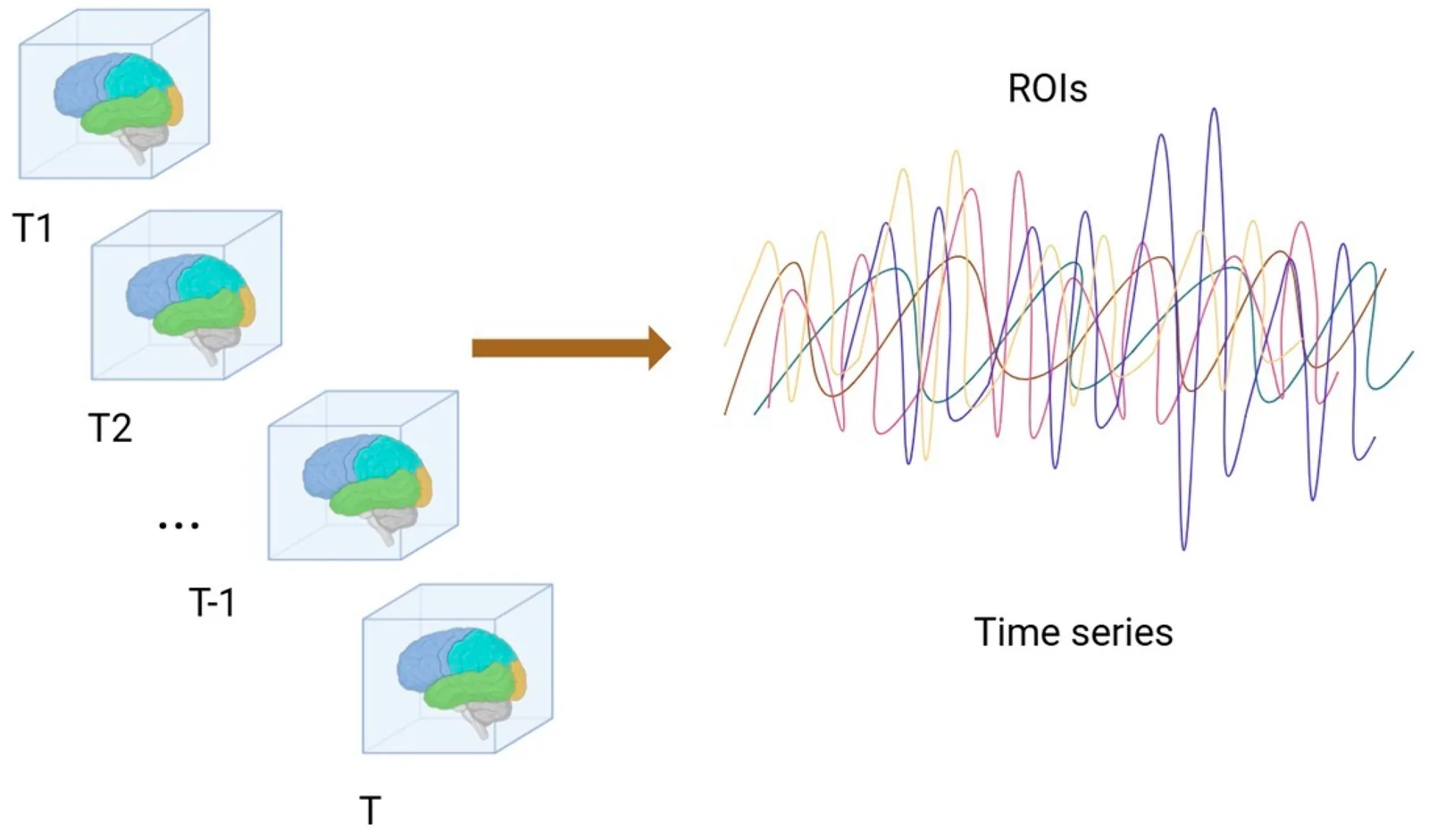
The extraction of time series.

fMRI data are four-dimensional, consisting of three-dimensional brain image information and one-dimensional time information. The extraction of time series is to convert four-dimensional data into a two-dimensional N × T matrix M. N is the number of cerebral regions, T denotes the temporal dimension, and Mij is the characteristic value of brain region i at time j. Therefore, fMRI data can be transformed into temporal sequences corresponding to individual brain regions. The schematic illustration of time series extraction is presented in Figure 1.

(3) Calculation of the Indexes of Brain Functional Connectivity

The commonly used coefficients to measure the relationship between brain regions include the Pearson correlation coefficient, partial correlation coefficient and wavelet coefficient. To quantify functional connectivity between ROIs, this study adopts the Pearson correlation coefficient as a simple yet widely used correlation metric. The formula of the Pearson correlation coefficient is as follows:

(1) $$\rho_{ij}=corr(x_{i},x_{j})=\frac{\sum_{t=1}^{N}\left(x_{jt}-\bar{x_{j}})(x_{it}-\bar{x_{i}}\right)}{\sqrt{\sum_{t=1}^{N}({\left(x_{it}-\bar{x_{i}}\right)}^{2})\sum_{t=1}^{N}{\left(x_{jt}-\bar{x_{j}}\right)}^{2}}}$$

In the above formula, $x_{i}$ and $x_{j}$ refer to the time series of the ROI i and ROI j, respectively, $\bar{x_{i}}$ and $\bar{x_{j}}$ represent the time series mean of the ROI i and ROI j. $\rho_{ij}$ denotes the Pearson correlation computed between a pair of ROIs, with a range of −1 ≤ $\rho_{ij}$≤ 1. If $\rho_{ij}$ is positive, it indicates a positive correlation between the signal intensities of a pair of ROIs over time, suggesting functional synergy. In contrast, a negative ρij points to functional antagonism across the ROIs, with a stronger correlation as the absolute value increases. When $\rho_{ij}$= 0, it can be concluded that there is no correlation between the signal intensities of the two ROIs over time. The result is a Pearson correlation matrix, where each column or row corresponds to the connectivity profile between a given ROI and all remaining ROIs.

## 3. Methods

### 3.1. Preliminaries

In modern SSM-based models, such as structured state space sequence models (S4) and Mamba [33,34,35], both rely on classical continuous systems that represent one-dimensional input functions or sequences as $x_{t}\in R$, mapped to the output $h_{t}\in R^{N}$ through an intermediate implicit state $y_{t}\in R$. The above procedure can be expressed by a linear ordinary differential equation (ODE) as:

(2) $$\dot{h_{t}}=Ah_{t}+Bx_{t},$$

(3) $$y_{t}=Ch_{t},$$

where $A\in R^{N\times N}$ is defined as the state matrix, with $B\in R^{N\times 1}$ and $C\in R^{N\times 1}$ serving as the projection parameters. To enhance compatibility with deep learning applications, S4 and Mamba implement discretization on this continuous system. Specifically, they integrate a temporal scaling factor ∆ and adopt a predetermined discretization method to convert the continuous matrices A and B into their discretized versions $\bar{A}$ and $\bar{B}$. As a common practice, the ZOH (zero-order hold) technique is employed to perform discretization, which can be formulated as:

(4) $$\bar{A}=e^{△A},$$

(5) $$\bar{B}={\left(△A\right)}^{-1}(e^{△A}-I)△B,$$

Once discretized, the SSM-based model can be computed using linear recursion:

(6) $$h_{t}=\bar{A}h_{t-1}+\bar{B}x_{t},$$

(7) $$y_{t}=Ch_{t},$$

Alternatively, the model can be expressed as a global convolution:

(8) $$\bar{K}=(C\bar{B},C\bar{AB},\ldots C{\bar{A}}^{L-1}\bar{B}),$$

(9) $$y=\bar{K}\times x,$$

where $\bar{K}\in R^{L}$ represents a structured convolution filter, and L is the size of the input sequence x.

Recently, Mamba2 introduced the SSD framework, which reduces matrix A to a scalar form [36]. Mamba2 is designed for efficient long-context modeling. Mamba2 operates using a structured state space representation. This special case of SSMs is applicable to both linear and quadratic scenarios. The mathematical formulation of Mamba2 (SSD) is presented as follows:

(10) $$y(t)=\sum_{i=1}^{t}C_{t}^{T}A_{t:i+1}B_{i}x(i),$$

(11) $$A_{t:i}=\prod_{j=i+1}^{t}A_{j},$$

where t (1 ≤ t ≤ T) denotes the current time step, while $A_{i}$,$B_{i}$,$C_{t}^{T}$ are dynamically updated parameterized matrices.

### 3.2. Architecture Overview

**Figure 2:**
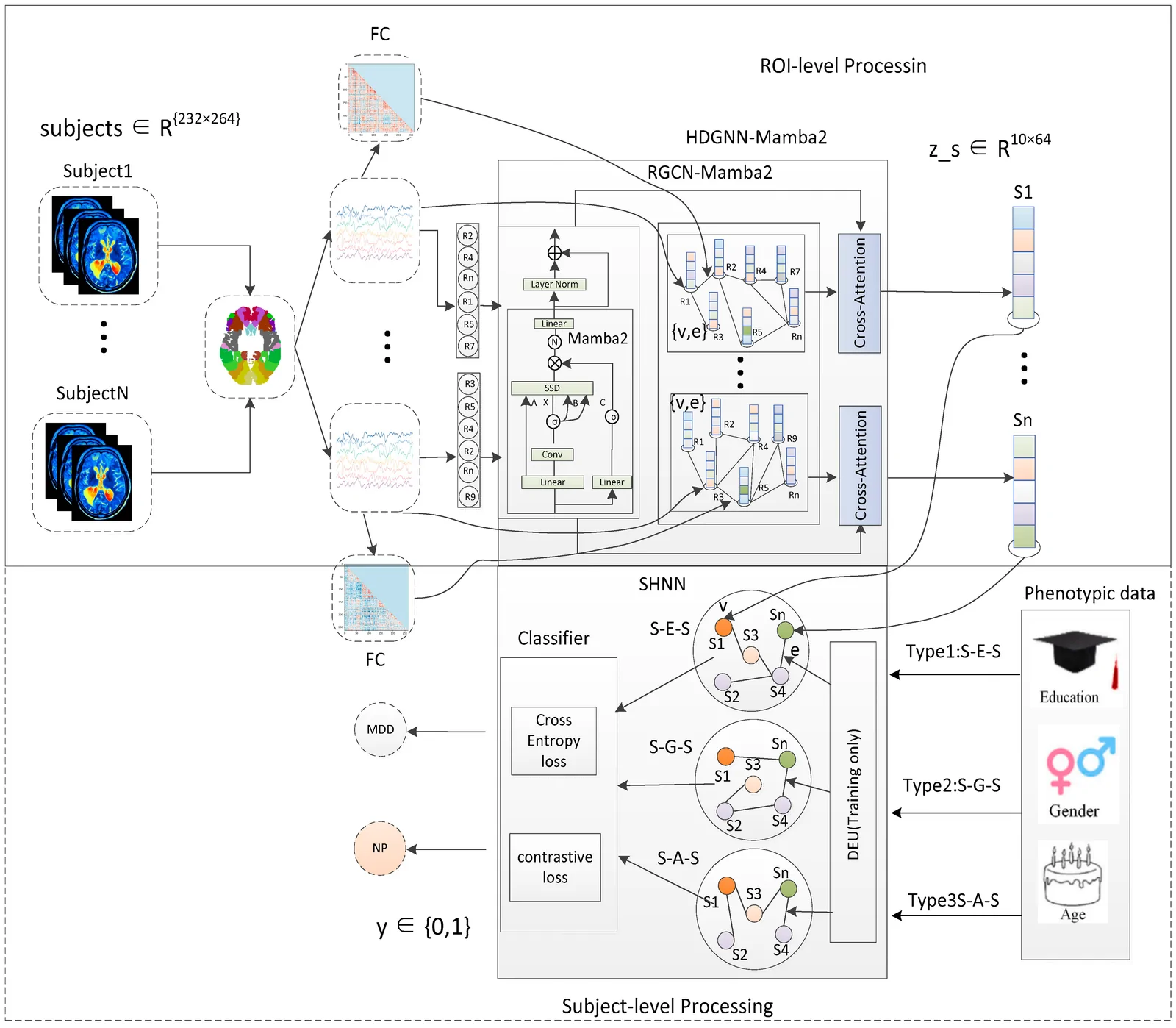
The overall architecture.

The HDGNN-Mamba2 framework is schematically depicted in Figure 2. The fMRI data used in this study were derived from the REST-meta-MDD dataset. For each individual subject, the preprocessed fMRI data were used to extract blood-oxygen-level-dependent average signal sequences corresponding to ROIs, defined according to a brain atlas template. We performed pairwise Pearson correlation analysis on the time series to construct functional connectivity matrices, which served as adjacency matrices for individual brain region graphs to characterize functional associations between pairwise brain regions. Simultaneously, the time series and the resulting adjacency matrices were input into the Mamba2-GNN block of the HDGNN-Mamba2 module, where bidirectional interaction between temporal and spatial features was achieved via a cross-attention fusion mechanism to learn spatiotemporal features of individual brain networks. The updated ROI regions of each individual brain area aggregated information from adjacent nodes. Three phenotypic datasets were processed using clustering methods to form three types of connectivity relationships among subjects, which were then used as edge attributes in the heterogeneous graph. Individual brain networks aggregated information from different types of edges through the HGNN layer of the HDGNN-Mamba2 module, obtaining heterogeneous global graph network feature embeddings that integrated multiple feature categories. Finally, the aggregated multimodal features were fed into a classifier to complete the prediction task. The HDGNN-Mamba2 was mainly composed of the following parts: a Mamba2-GNN block, an HGNN block and a classifier layer.

### 3.3. Mamba2-GNN

**Figure 3:**
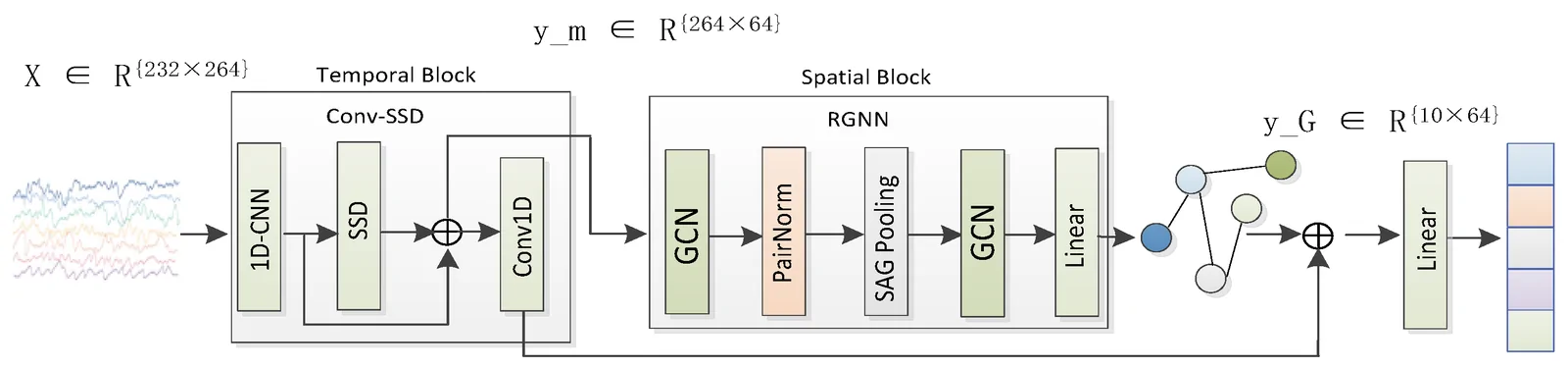
The architecture of the Mamba2-GNN block.

MDD affects different etiological brain regions, which implies that not all brain regions hold equal importance in diagnosing MDD. As shown in Figure 3, we proposed the Mamba2-GNN block method to extract spatiotemporal features of individual brain regions. This method primarily consisted of the Temporal Block and Spatial Block, eliminating noisy connections while preserving critical vertices and edge linkages.

#### 3.3.1. Temporal Block

As illustrated in Figure 3, the temporal block used a Conv-SSD layer to capture temporal dependencies in fMRI series. The core module of the Conv-SSD layer was Mamba2, which was equipped with a 1D convolutional layer (1D-CNN), layer normalization, and residual connections that improved training stability and overall performance. First, a 1D convolutional layer was adopted to encode the initial fMRI time series x into a hidden state x^′^. Second, within the Conv-SSD layer, this component processed the full $x^{\prime }$ sequence to extract features. The feature data were then divided into two streams: one stream was fed as input $X_{R}$ to the Spatial Block, while the other was processed through dimensionality reduction using a larger 1D convolutional kernel (3 × 3). The result was then fused with the Spatial Block’s output through a residual connection. The Conv-SSD layer was defined as:

(12) $$x^{\prime }=Conv1d(x),$$

(13) $$X_{R}=SSD(x^{\prime },A,B,C,∆)+x^{\prime },$$

(14) $$y_{m}=Conv1d(X_{R}),$$

The Conv-SSD layer mapped the input time series to a hidden representation with a unified dimension *D* = 64. Thus, the output temporal feature was $y_{m}$ ∈ R*^N^* ^× *D*^, where *N* = 264.

#### 3.3.2. Adjacency Matrix

Individual brain networks carry topological information, and graph neural networks exhibit significant advantages in processing such node-edge connection data.

Consequently, each subject was modeled as a graph $G_{R}=(V,E)$, in which V signified the fMRI temporal feature data of each ROI extracted subsequent to the Conv-SSD layer, and E was the edge weights capturing the interconnection patterns between nodes. The Pearson correlation coefficient $\rho_{ij}$ computed in the prior section was utilized in this context. The adjacency matrix $A_{ij}$ was shown below.

(15) $$A_{ij}=\left\{\begin{array}{c} {|\rho }_{ij}|\;,\;\mathrm{if}\;i\neq j\\ 1\;,\;\mathrm{if}\;i=j \end{array}\right.,$$

where all pairs of ROIs were fully connected, with edge weights given by the absolute Pearson correlation coefficient ${|\rho }_{ij}|$ to quantify functional associations between brain regions. The adjacency matrix was denoted as $A_{ij}\in R^{N\times N}$, where *N* = 264. A fully connected graph was adopted to avoid arbitrary thresholding that may discard weak but potentially informative functional relationships.

#### 3.3.3. Spatial Block

The core module in the Spatial Block was the graph neural network module for brain region ROIs (RGNN). The RGNN was primarily composed of ROI graph convolutional layers (GCN), normalization layers (PairNorm), pooling layers (SAGPooling), and others. The calculation formula for one graph convolutional layer and normalization layer was as follows:

(16) $$X_{R}^{l+1}=\sigma (GCN(X_{R},A_{R}))=\sigma ({\dot{D}}_{R}^{-1/2}\cdot {\dot{A}}_{R}\cdot {\dot{D}}_{R}^{-1/2}\cdot X_{R}^{l}\cdot W_{R}^{l}),$$

(17) $${X_{R}^{l+1}}^{\prime }=Norm(X_{R}^{l+1}),$$

where R is the input graph, $W_{R}^{l}$ denotes the learnable weight matrix, ${\dot{A}}_{R}=A_{R}+I$ is the adjacency matrix with added self-loops, $D_{R}$ is the degree matrix, ${\dot{D}}_{R}^{-1/2}\cdot {\dot{A}}_{R}\cdot {\dot{D}}_{R}^{1/2}$ represents the normalized Laplacian matrix, σ represents the activation function, with $X_{R}^{l}$ and $X_{R}^{l+1}$ respectively denoting the node embeddings learned at layers l and l + 1. The normalized Laplacian operator implicitly downweights weak edges during spectral convolution, mitigating noise amplification in the fully connected graph.

Empirical findings suggest that under specific circumstances, the classification efficacy of Graph Neural Networks (GNNs) may not exhibit improvement with the augmentation of layer depth; rather, it can experience notable degradation. This phenomenon is associated with the potential over-smoothing challenge that may emerge in GNN architectures. Within the model proposed in this work, the node normalization layer was designed to normalize node representations, avoiding the convergence of all node embeddings to a similar state. This mechanism not only improved the stability of GNNs but also contributed to alleviating overfitting. The detailed implementation procedures were outlined as follows:

First, we centralized each node’s features to obtain:

(18) $${\bar{h}}_{i}^{l+1}=h_{i}^{l+1}-\frac{1}{n}\sum_{i=1}^{n}h_{i}^{l+1},$$

Let s be the scaling factor; then ${\bar{h}}_{i}^{l+1}$ was scaled to yield:

(19) $${\overset{̿}{h}}_{i}^{l+1}=s\cdot (\frac{{\bar{h}}_{i}^{l+1}}{\sqrt{\frac{1}{n}\sum_{i=1}^{n}||{\bar{h}}_{i}^{l+1}{\left|\right|}_{2}}}),$$

By substituting $\sum {\left||{\bar{h}}_{i}^{l+1}|\right|}_{2}$ in the preceding expression with $n\sum {\left||{\bar{h}}_{i}^{l+1}|\right|}_{2}$, the PairNorm in SI (scaling individually) mode was obtained. Thus, the equation became:

(20) $${\overset{̿}{h}}_{i}^{l+1}=s\cdot (\frac{{\bar{h}}_{i}^{l+1}}{{\left||{\bar{h}}_{i}^{l+1}|\right|}_{2}}),$$

While the graph was fully connected at initialization, adaptive sparsification was achieved during training by the SAGPooling module, which retained only the top-K most discriminative nodes. A pooling module driven by self-attention mechanisms (SAGPooling) was devised to extract node features with discriminative power alongside selecting salient nodes. A node-wise pooling component was employed to coarsen the graph, retaining only the most important nodes. Furthermore, the count of network parameters could be decreased by the pooling layer, thereby enabling overfitting to be mitigated. In this manner, higher-level graph structural representations could be conveyed by the node representations generated on the coarsened graph.

Let the node features of the current layer be denoted by $X_{R}^{l}$, and let the self-attention score matrix $S_{R}^{l}$ be estimated through graph convolution. A Top-K node screening mechanism was then employed to retain a selection of nodes from the local brain, with k denoting the node count required to construct a new graph. When selecting K nodes, the node ranking operation was based on indices idx of the top K values output by $S_{R}^{l}$. Subsequently, by utilizing the tanh activation function across the top K component $S_{R}^{l}$, the self-attention weights were derived. Then, via a point-wise product between $X_{R}^{l}$ and s, the pooled node features $X_{R}^{l+1}$ were obtained. The adjacency matrix $A_{idxl,idxl}^{l}$ underwent row and column operations to construct the new adjacency matrix $A_{R}^{l+1}$.

(21) $$S_{R}^{l}=GCN(X_{R}^{l},A_{R}),$$

(22) $$s=tanh(S_{R}^{l}),$$

(23) $${idx}_{R}^{l}={TOP}_{k}(s),$$

(24) $$X_{R}^{l+1}=X_{idxl}^{l}\cdot s,$$

(25) $$A_{R}^{l+1}=A_{idxl,idxl}^{l},$$

The final output $y_{G}=[y_{g1},y_{g2},\ldots ,y_{gk}]$ of the Spatial Block was generated by further applying RGNN layers to K chosen node attributes.

(26) $$y_{G}=Linear(GCN(X_{R}^{l+1})),$$

A cross-attention fusion mechanism was proposed that enabled bidirectional interaction between temporal and spatial features. The motivation was that the K brain region activities were inherently spatiotemporal, where the activity of a region at a given time was influenced by both its own temporal dynamics and its topological connections to other regions. Furthermore, temporal and spatial information provided complementary perspectives on brain function, and their interaction can reveal patterns that neither modality captured alone.

(27) $${y^{\prime }}_{G}=CrossAtten(y_{G},y_{m,k})+y_{G},$$

(28) $${y^{\prime }}_{m}=CrossAtten(y_{m,k},y_{G})+y_{m,k},$$

where CrossAttn(A,B) denotes the standard cross-attention mechanism with A as the query and B supplying the value and key. $y_{m,k}$ denote temporal features of the k brain regions retained in the Spatial Block, extracted from $y_{m}$ using the same index set idx. The final output Y of the Mamba2-GNN layer was adaptively fused through a learnable gating mechanism:

(29) $$Y=g_{GM}\cdot {y^{\prime }}_{m}+(1-g_{GM}){y^{\prime }}_{G},$$

where $g_{GM}$ was a gating vector that dynamically balanced the contribution of temporal and spatial information for each brain region.

Then the individual brain region features were reshaped into a 1D vector, which served as the node feature for the subsequent heterogeneous graph. In this graph, every subject was treated as a node, and the flattened feature vector captured the subject’s discriminative spatiotemporal brain patterns.

### 3.4. HGNN

Previous studies rarely considered the differences in patient demographic data. To reflect these differences, we constructed a heterogeneous population relationship graph (HGNN) Gs = {V’,E’}, for all of the subjects S = {S1, S2,…, Sn}, based on demographic data and fMRI data. Here, n is the total number of subjects, E’ defines the edges connecting vertices, and the node set V’ represents the subjects. Diverse demographic data (e.g., sex, age, educational attainment) served as edge relations, which were determined using clustering methodologies.

Taking subject sex as an example, clustering yielded groups of the same sex, where the same-sex relationship was assigned a value of 1 and different-sex relationships were assigned 0, forming an adjacency matrix. Ultimately, a heterogeneous graph structure with different path sets θ={S−G−S;S−E−S;S−A−S} was constructed, where S represents subjects, S-G-S denotes sex-related paths between subjects, S-E-S represents education-related paths between subjects, and S-A-S indicates age-related paths between subjects. Heterogeneous graph node features $h=\{Y_{1},Y_{2},\ldots ,Y_{D}\}\in R^{n\times m}$, where m = k × D is the feature dimension. The adjacency matrix of V’ is $A_{\theta }^{0}$. $A_{\theta }^{0}\in R^{n\times n}$ represents the clustering-based relationship scores between two subjects derived from demographic data. Age and education were discretized via k-means (k = 2, Euclidean distance) fitted on the training set within each fold; the resulting binary groupings were refined by dynamic edge updating.

To further enhance the expressive power of the heterogeneous graph, a Dynamic Edge Updating (DEU) mechanism was introduced that periodically refined the inter-subject adjacency matrices during training. For each phenotypic path θ, the dynamic similarity was fused with the original static edge using a learnable gate $g_{\theta }$. The fused adjacency matrix ${A^{\prime }}_{\theta }$ was as follows:

(30) $${A^{\prime }}_{\theta }=g_{\theta }\cdot S_{h}+(1-g_{\theta })\cdot A_{\theta }^{0},$$

where $S_{h}$ is the cosine similarity matrix computed from L2-normalized node embeddings, with values in [−1, 1] and no thresholding applied. To ensure training stability, the edge updates were performed once every Δ = 10 epochs, and the adjacency matrix remained constant between updates. Self-edges were preserved, and the scalar gate g_θ_ was learnable and differentiable.

**Figure 4:**
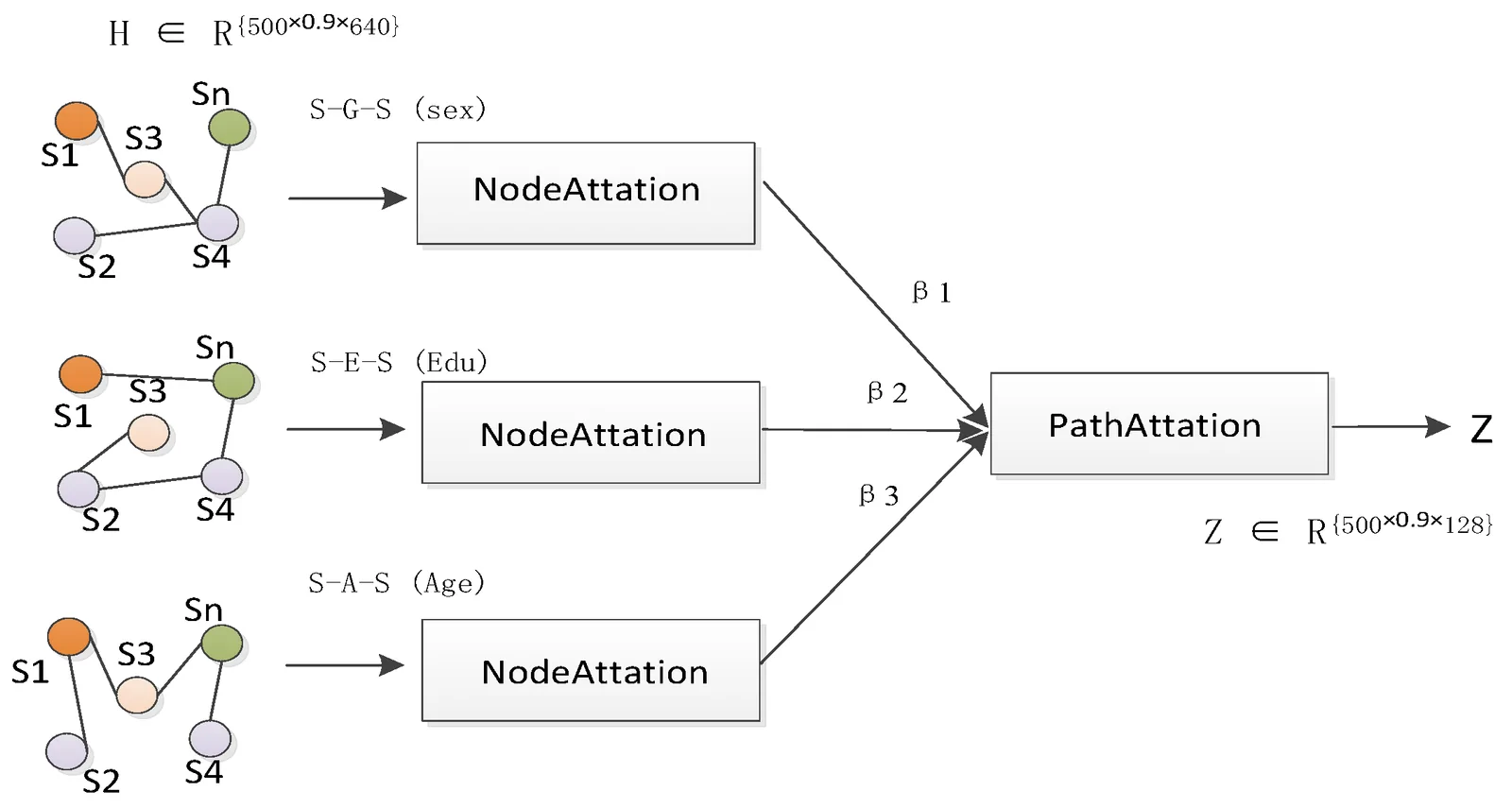
The architecture of the HGNN layer.

HGNN layer primarily employed an attention-based graph convolutional module as the backbone network for feature learning. The schematic diagram of the architecture of HGNN is shown in Figure 4. The layer first computed intra-path graph aggregation by determining the attention weight $\alpha_{ij}^{\theta }$ from node i to node j. Once the attention weights between nodes were computed, message aggregation was performed on the neighbors of node to aggregate information from different paths and neighbors, thereby obtaining the graph feature $z_{i}^{\theta }$ for each path. Subsequently, all path information was adaptively aggregated through learnable proportional weights for each path. The formula was as follows:

(31) $$H^{\prime }={A^{\prime }}_{\theta }\cdot H,$$

(32) $$e_{ij}^{\theta }={att}_{node}(H_{i}’,H_{j}’;\theta ),$$

(33) $$\alpha_{ij}^{\theta }=softmax(e_{ij}^{\theta }),$$

(34) ziθ=σk‖(∑j∈Nearαijθ·hj’),

(35) $$w_{\theta }=\frac{1}{\left|\theta \right|}\sum_{i\in |\theta |}q^{T}\cdot tanh(W\cdot z_{i}^{\theta }+b),$$

(36) $$\beta_{\theta }=softmax(w_{\theta }),$$

(37) $$Z=\sum \beta_{\theta }\cdot z_{\theta },$$

$\alpha_{ij}^{\theta }$ represents the normalized calculation of node weights $e_{ij}^{\theta }$, $w_{\theta }$ denotes the weight of each path, $\beta_{\theta }$ is the normalization of $w_{\theta }$, $q^{T}$ is the learnable parameter for path types, W and b are learnable parameters, ‖ k denotes multi-head attention, and |θ| represents the total number of paths.

### 3.5. Heterogeneous Graph Supervised Contrastive Learning

To further boost the discriminative capacity of the subject embeddings learned by the heterogeneous graph, a supervised contrastive learning module tailored for brain disorder diagnosis was introduced. Positive and negative pairs were constructed based on clinical diagnostic labels, requiring no additional data preprocessing or augmentation.

Z = {z1, z2, …, zN} is the set of subject embeddings output by the HGNN module. Each subject i is associated with a diagnostic label $\tilde{y}$ ∈ {0, 1}, where 0 denotes HC and 1 denotes MDD. The positive mask matrix Pij and negative mask matrix $N_{ij}$ are defined as:

(38) $$P_{ij}=\left\{\begin{array}{c} 1,\;\mathrm{if}\;{\tilde{y}}_{i}={\tilde{y}}_{j}\;\mathrm{and}\;i\neq j\\ 0,\;\mathrm{otherwise} \end{array}\right.,$$

(39) $$N_{ij}=\left\{\begin{array}{c} 1,\;\mathrm{if}\;{\tilde{y}}_{i}\neq {\tilde{y}}_{j}\;\\ 0,\;\mathrm{otherwise} \end{array}\right.,$$

The similarity between any pair of subject features was computed:

(40) $$sim(z_{i},z_{j})=\frac{z_{i}^{T}z_{j}}{{\left|z_{i}\right|}_{2}\cdot {\left|z_{j}\right|}_{2}},$$

For each subject i, the contrastive loss was defined as:

(41) $$L_{cl}^{i}=-log\frac{\sum_{j=1}^{N}P_{ij}\cdot exp(sim(z_{i},z_{j})/\tau )}{\sum_{k\neq i}^{N}exp(sim(z_{i},z_{k})/\tau )},$$

where τ > 0 is a temperature parameter that adjusts the smoothness of the similarity distribution. A smaller τ amplifies the differences between similarities, leading to a harder assignment. The embeddings used for metric learning were cached at the beginning of each epoch via a gradient-free forward pass and detached from the computation graph. This allowed the metric-learning objective to be computed over the entire training fold, providing sufficient non-anchor samples for stable optimization despite the small batch size, while preventing gradient backpropagation through cached embeddings and restricting updates to the current mini-batch. The cache was refreshed each epoch because subject representations evolved during training. Test-fold subjects were explicitly excluded from this cache.

The total loss combined the classification loss and the contrastive loss:

(42) $$L_{cl}=\frac{1}{N}\sum_{i=1}^{N}L_{cl}^{i},$$

By minimizing this loss, the model learned to pull embeddings of same-class subjects closer and push embeddings of different-class subjects apart.

### 3.6. Classification and Loss Function

The feature vector Z = {z_1_, z_2_, …, z_N_} output by the HGNN layer was passed to a fully connected layer. The fully connected layer output features for the cross-entropy loss, defined as:

(43) $$o_{j}=softmax(W\cdot z_{j}+b),$$

(44) $$L_{ce}=-\frac{1}{N}\sum_{j=1}^{N}\left[{\tilde{y}}_{j}log(o_{j})+(1-{\tilde{y}}_{j})log(1-o_{j})\right],$$

where $\tilde{y}$ ∈ {0, 1} is a labeled sample in the fMRI data.

The overall loss of the HDGNN-Mamba2 framework was a weighted sum of the contrastive loss and the cross-entropy classification loss:

(45) $$L=\lambda \cdot L_{cl}+L_{ce},$$

where λ > 0 was a balancing hyperparameter that controlled the relative contribution of the contrastive loss.

## 4. Experiments and Results

### 4.1. Training Setup

In the paper, a ten-fold stratified cross-validation framework was adopted to measure the algorithm’s performance. Following dataset shuffling, the entire data corpus was partitioned into ten sub-datasets. For each validation iteration, one sub-dataset served as the test set, while the other nine sub-datasets were merged to create the outer training set. Within each outer training set, a stratified inner split was performed to reserve 10% as an internal validation subset for early stopping, with the remaining 90% used as the training inner subset for model parameter updates. To prevent information leakage, all data-dependent operations—including clustering for phenotypic attributes, graph adjacency reconstruction, dynamic edge updating, contrastive pair generation, and normalization parameters—were confined exclusively to the training inner subset within each cross-validation fold. The internal validation subset was used only for monitoring validation loss during early stopping and was not involved in graph construction, clustering, or parameter updates. Specifically, the k-means centers for age and education were fitted exclusively on the training inner subset and then applied to discretize the held-out test dataset. Both individual brain graph adjacency matrices and the heterogeneous subject graph were constructed using only training inner subset subjects, with test subjects subsequently embedded via inductive inference using the fixed training-derived graph structure and node parameters. All normalization parameters were computed solely from the training inner subset and applied to the validation and test datasets without re-estimation. The cosine similarity matrix and learnable gates for dynamic edge updating were derived exclusively from training inner subset embeddings, with validation and test subjects excluded from the computation. For supervised contrastive learning, the gradient-free forward pass and embedding cache were built from training inner subset subjects only, and positive and negative pairs were formed using training diagnostic labels alone, ensuring that the test dataset remained entirely isolated until final evaluation. The complete inductive procedure is formalized in Algorithm 1.
**Algorithm 1:** Per-Fold Inductive Cross-Validation for HDGNN-Mamba2Input: Dataset D with *n* = 533 subjects, 10-fold stratified splitOutput: Mean ± Std of metrics across 10 foldsPartition 533 subjects into 10 stratified foldsfor each fold k = 1 to 10 do  D_train ← 9 folds (~480 subjects), D_test ← 1 fold (~53 subjects)  D_train_s, D_val ← stratified_split(D_train, ratio=0.9/0.1)   // ── Stage 1: Preprocessing  C_age = k-means(D_train_s.age, k=2)  C_edu= k-means(D_train_s.edu, k=2)  // Build adjacency matrix: TRAIN nodes only  A_train←{Samesex(D_train_s.sex),SameCluster(D_train_s.edu,C_edu), SameCluster(D_train_s.age, C_age)}  //S-G-S,S-E-S,S-A-S  // ── Stage 2: End-to-End Training (HDGNN-Mamba2)  for epoch = 1 to 100 do      for each subject s in D_train_s do          z_s ← Mamba2_GNN({X_s})      end for      in_emb_train ← {z_s | s ∈ D_train_s}      //Dynamic edge update (every Δ epochs, train embeddings only)      A^′^_train ← DEU( A_train, sim( in_emb_train ), Δ=10 ) // Dynamic edge update, train only      Z_train = HGNN( A^′^_train, {in_emb_train} )    // Population graph, train only      Z_cache ← Detach(Z_train)        // Training inner subset, detached      L_cl ← SupervisedcontrastiveLoss(Z_cache, D_train_s.y) // Standard supervised contrastive loss      L_ce ← CrossEntropy(Z_train, D_train_s.y)      L ← L_ce + λ·L_cl       L.backward(); optimizer.step()      val_loss, val_acc ← Evaluate(D_val)       if val_loss not improved for 20 epochs then          break       end if       RestoreBestModelState()  end for  //── Stage 3: Test Evaluation  // Build test adjacency: test nodes connect to train nodes via phenotype  A_test ←∅  for each test subject t in D_test do      z_t ← Mamba2_GNN(X_t)  // Test subject feature  // Connect test node to top-K train nodes by phenotype similarity      sim_train ← Similarity(t, D_train_s, [sex, age, edu])      top_k ← TopK(sim_train, k=5)      A_test ← A_test ∪ {(t, neighbor) | neighbor ∈ top_k}  end for  // Concatenate train and test, forward through HGNN (no grad)  Z_all ← HGNN_Inference(A_train ⋃A_test, concat(in_emb_train, {z_t}))  ŷ_test ← argmax(Z_all[n_train_s:])  // Test predictions    Compute metrics: ACC, SEN, SPE, AUROC, AUPRCend forreturn Mean ± Std of metrics across 10 folds

The detailed model configuration is as follows. The Mamba2-GNN block contains two temporal Conv-SSD layers and two RGNN spatial layers, both with a unified hidden dimension of 64. The SAGPooling layer retains the top K = 10 nodes (pooling ratio ≈ 0.038) and used four attention heads. The HGNN block comprises two attention-based graph convolutional layers with a hidden dimension of 64. The random seed was set to 42. All experiments were implemented using PyTorch 2.0.1 and PyTorch Geometric 2.3.1 on a Linux operating system with an NVIDIA RTX 4090 GPU (24 GB memory).

The training hyperparameters were set as follows: a learning rate of 0.0001, a dropout rate of 0.5, and a weight decay of 0.0001, following conventional settings in deep learning for small-sample neuroimaging classification. For the supervised contrastive learning module, τ = 0.5, Δ = 10, and λ = 0.1 were selected based on commonly adopted values in contrastive learning for graph-structured data and were fixed a priori without dataset-specific tuning on the S20. The pooling ratio (K = 10) was predetermined for clinical interpretability and not treated as a tunable hyperparameter. The Adam optimizer was used with a batch size of 4. Early stopping was monitored on a 10% stratified validation subset held out from each fold’s training set.

### 4.2. Statistical Metrics

For model evaluation, precision, accuracy (ACC), F1-score, and Recall served as the key metrics. First, it is necessary to clarify four concepts: TN (true negative), TP (true positive), FP (false positive), and FN (false negative). TP denotes the number of MDD subjects correctly classified as MDD. TN denotes the number of healthy controls correctly classified. FP denotes the number of healthy controls mistakenly classified as MDD. FN denotes the number of MDD subjects mistakenly classified as healthy controls.

These metrics are defined as follows:

(46) $$Accuracy=\frac{TN+TP}{TP+FP+FN+TN},$$

(47) $$Precision=\frac{TP}{FP+TP},$$

(48) $$Recall=\frac{TP}{TP+FN},$$

(49) $$F1=2\times \frac{Precision\times Recall}{Precision+Recall},$$

In binary classification, Sensitivity (also termed Recall) is defined as TP/(TP + FN), and Specificity as TN/(TN + FP). Precision is equivalent to Positive Predictive Value (PPV), defined as TP/(TP + FP).

### 4.3. Classification of fMRI Data

**Figure 5:**
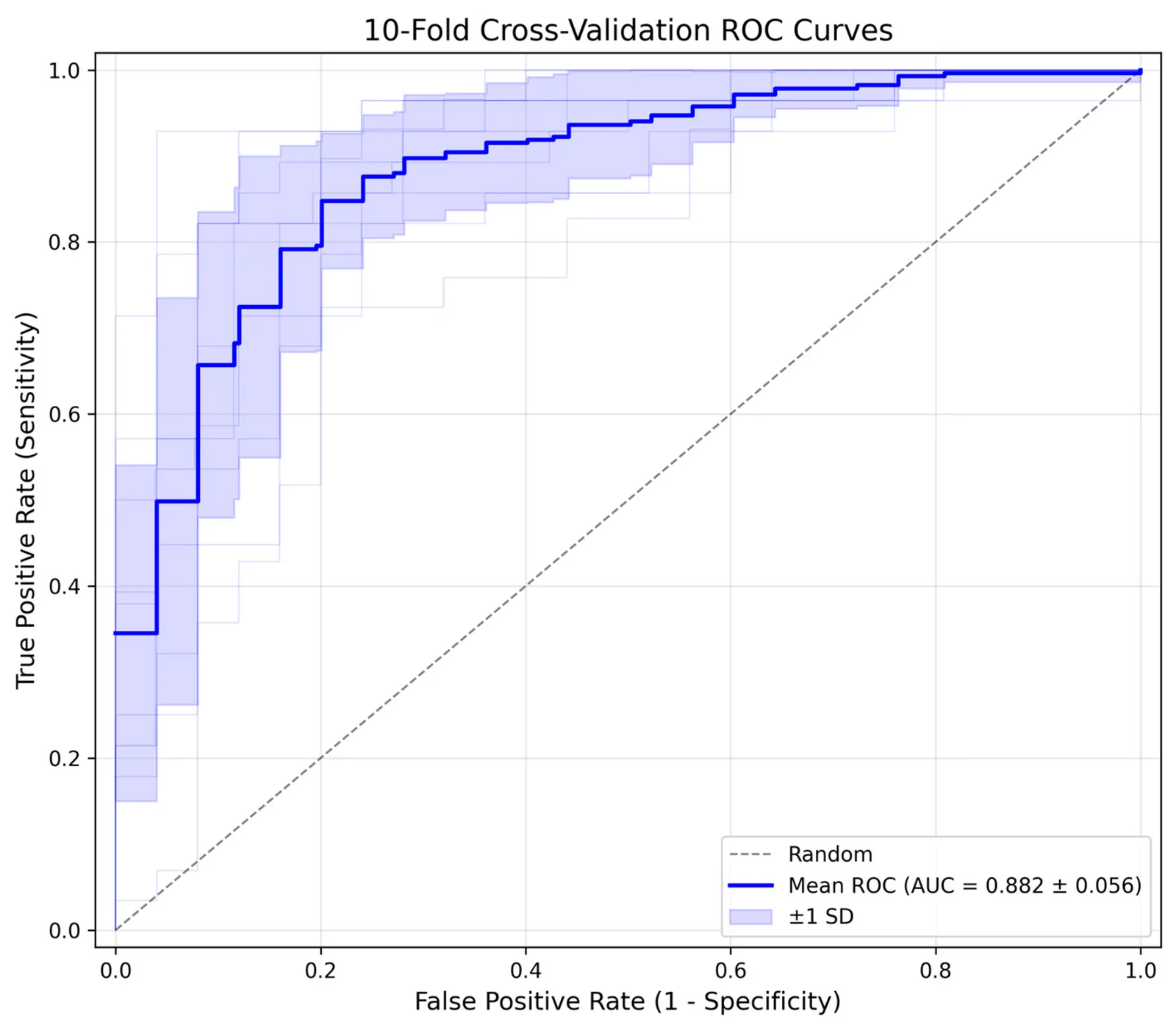
Ten-fold averaged ROC curves.

**Figure 6:**
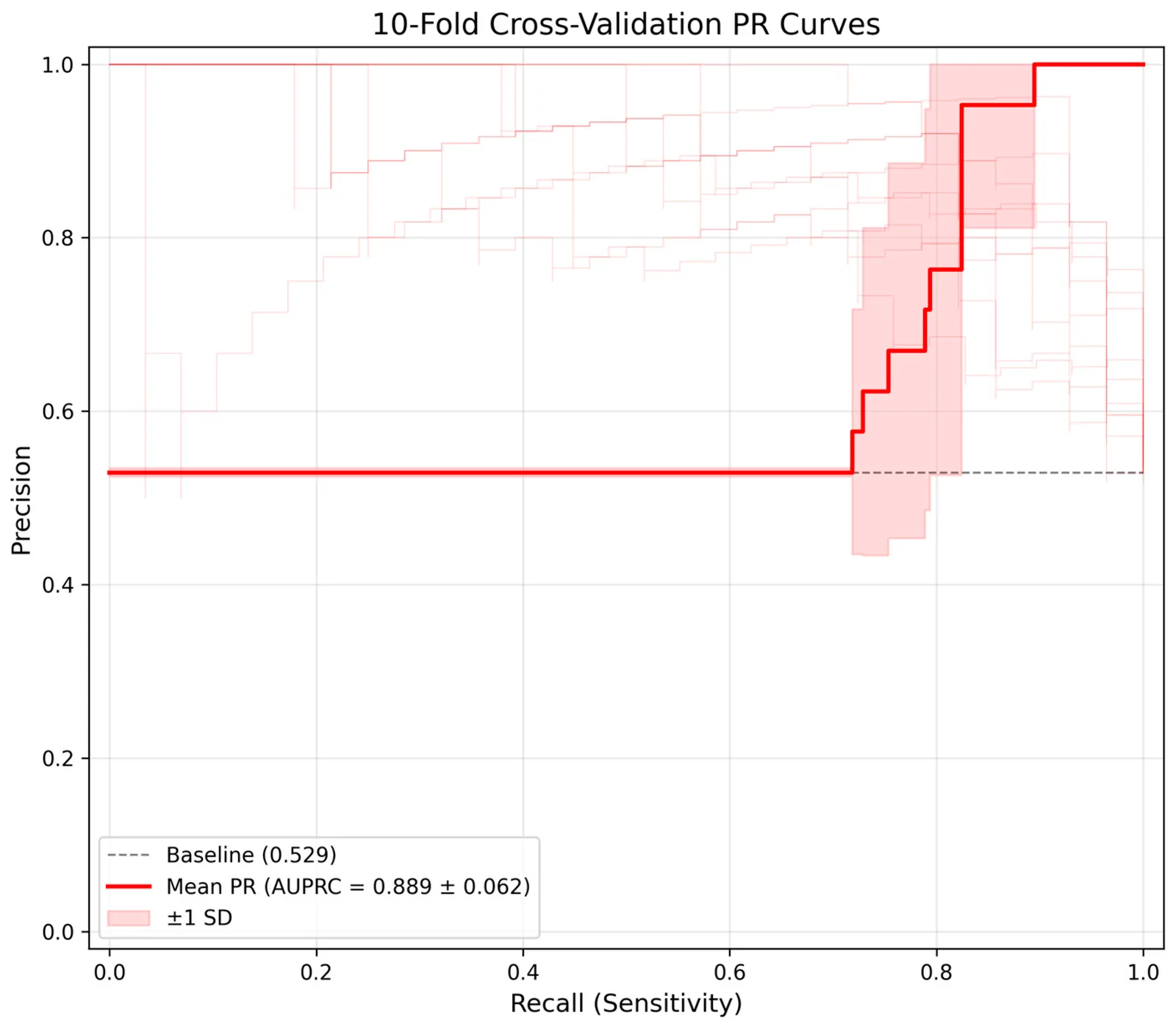
Ten-fold averaged precision-recall curves.

The HDGNN-Mamba2 model achieved a mean accuracy of 0.8388 ± 0.0542 for MDD classification under the inductive evaluation protocol. The discriminative performance of HDGNN-Mamba2 across all ten folds is summarized in Figure 5 and Figure 6. Figure 5 presents the ten-fold averaged ROC curve, with a mean AUROC of 0.8819 ± 0.0565. Figure 6 presents the ten-fold averaged precision–recall curve, with a mean AUPRC of 0.8886 ± 0.0622. The ROC curve consistently exceeds the random-guess diagonal, and the PR curve exceeds the random-guess baseline over a broad range of recall values. Regarding model complexity, the HDGNN-Mamba2 architecture contains approximately 0.46 million trainable parameters. The complete 10-fold cross-validation experiment required approximately 15 h and 8 GB peak memory. Detailed per-fold performance metrics are provided in Supplementary Table S1.

To verify the efficacy of the suggested approach, we carried out comparative experiments against the following methods. The REST-meta-MDD project dataset was used for all comparative experiments. The SVM model was implemented through Scikit-learn. The GCN and GAT models were implemented using the PyTorch Geometric library. The results of the comparison are shown in Table 2.

To statistically evaluate the accuracy difference between HDGNN-Mamba2 and MFGCN, we conducted a paired *t*-test on per-fold accuracy across ten folds. The mean difference was 0.30 percentage points (SD = 2.68, *p* = 0.73, 95% CI [−1.62%, +2.22%]), indicating that the two models achieve comparable accuracy on this dataset. HDGNN-Mamba2 trades a marginal F1-score reduction (0.8490 vs. 0.8528) for higher sensitivity (86.52% vs. 82.73%), reflecting a recall-oriented precision-recall trade-off. The distinct advantages of HDGNN-Mamba2 are its architectural innovations including cross-attention fusion, dynamic edge updating, and interpretable attention-based pooling.

### 4.4. Ablation Experiment

Ablation experiments were performed on the test dataset to verify the effectiveness of the proposed model and method. To evaluate the contribution of each module, we conducted ablation experiments by removing them individually and in combination. Specifically, we compared the full HDGNN-Mamba2 model against configurations without cross-attention fusion, without DEU, and without Supervised Contrastive Learning (λ = 0). Additionally, we compared against the original component-level baselines, including standalone Conv-SSD (Temporal only), Conv-SSD + HGNN (without Spatial), RGNN + HGNN (without Temporal), HGNN (without Mamba2-GNN), and Mamba2-GNN.

Ten-fold cross-validation was employed to assess the classification capability of each model on the dataset. The corresponding results are reported in Table 3.

The experimental results of the ablation conditions show that the full HDGNN-Mamba2 model achieves the highest classification performance. The final configuration yields 83.88% accuracy (ACC), 86.52% sensitivity (SEN), 80.85% specificity (SPE), 88.19% AUROC, and 88.86% AUPRC. The Mamba2-GNN configuration (0.8033) represents the imaging-only backbone—individual spatiotemporal brain network features without the heterogeneous demographic graph—enabling quantification of the incremental contribution of phenotypic information. The full model gains +3.55% over this imaging-only baseline, while the standalone HGNN (0.5629), relying solely on demographic graph structure without imaging features, achieves only chance-level performance.

The ablation results allow us to quantify the incremental contribution of each module. Removing the cross-attention fusion caused a 5.17% absolute drop in accuracy, indicating that bidirectional spatiotemporal interaction contributes substantially. Removing the dynamic edge updating mechanism reduced accuracy by 4.35%, confirming that phenotypic relationships provide complementary structural priors. Removing Supervised Contrastive Learning led to a 2.38% decrease, suggesting that the primary supervision signal comes from the cross-entropy loss, while the contrastive term acts as a beneficial regularizer. These observations collectively indicate that the performance gain is not driven by a single over-parameterized component but by the synergistic integration of complementary inductive biases.

### 4.5. Impact of Demographic Data

Within conventional machine learning approaches, when demographic variables display pronounced intergroup disparities, identifying the specific contributing factors underlying disparities in cerebral metrics becomes challenging. In such scenarios, it is necessary to regress out and eliminate intergroup-varying variables (e.g., sex, age) from physiological measurements (e.g., functional connectivity) to mitigate their influence. By contrast, deep neural network-based methods are data-centric and eliminate the need for manual feature extraction. Consequently, within categorization tasks, phenotypic information can be directly incorporated without engaging in handcrafted feature filtering procedures. As shown in Table 4, the results reveal that classification performance with integrated demographic data surpasses that of the non-integrated counterpart, thereby validating the efficacy of demographic variables within multi-level information fusion frameworks.

Importantly, the combination of all three phenotypic attributes (sex, age, and education) yields the highest accuracy, demonstrating that multimodal phenotypic information provides complementary benefits for MDD classification.

### 4.6. Analysis of Relevant Brain Regions

Within this section, we principally examine the regions of interest in the brain linked to depression. The parameter weights of each brain region produced by the self-attention pooling layer in HDGNN-Mamba2 are crucial for generating discriminative features.

First, fMRI data encompassing all ROIs were fed into the HDGNN-Mamba2 for forward propagation. Secondly, fMRI data extracted spatiotemporal features in the Mamba2-GNN layer, particularly acquiring spatial features through graph neural networks in the RGNN layer. Hierarchical graph dimensionality reduction was achieved through the self-attention TopK pooling operation, which compresses the graph structure while selecting K important nodes and retaining the information of these key nodes. These K nodes serve as the most discriminative regions for MDD classification. The value of K in this paper is set to 10. Finally, after multiple rounds of iterative model calculations, statistical analysis was performed on the top 10 important brain regions following TopK pooling, and the top ten brain regions with the highest selection frequency were identified as model-attributed regions.

**Figure 7:**
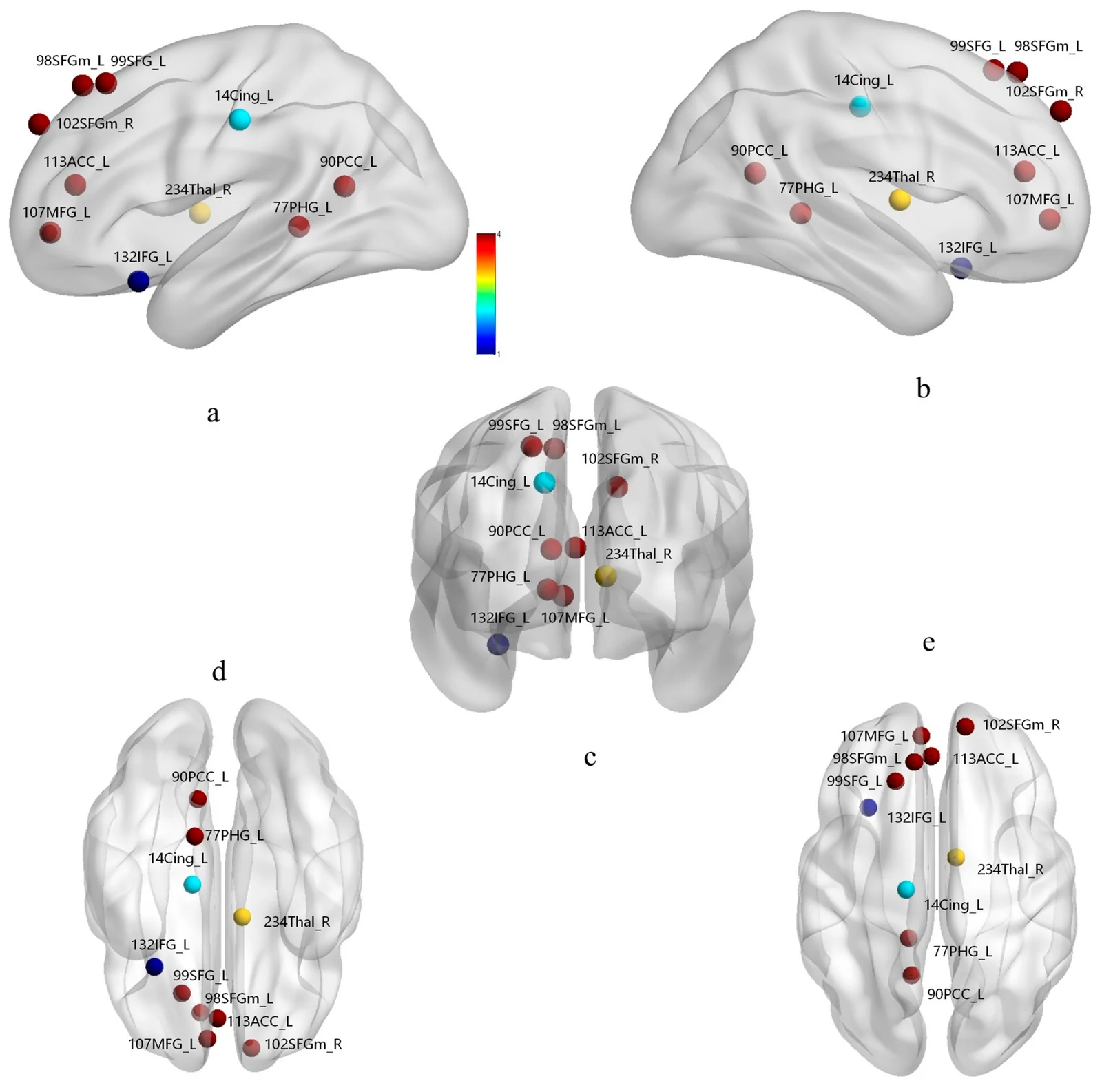
Illustration of relevant brain regions.

The top ten ROIs with the highest mean node pooling values are labeled as 77, 98, 113, 90, 14, 107, 132, 234, 99, and 102. These brain regions represent Parahippocampal Gyrus (limbic lobe), Superior Frontal Gyrus (frontal lobe), Anterior Cingulate (limbic lobe), Posterior Cingulate (limbic lobe), Cingulate Gyrus (limbic lobe), Medial Frontal Gyrus (frontal lobe), Inferior Frontal Gyrus (frontal lobe), Thalamus, Superior Frontal Gyrus (frontal lobe), Superior Frontal Gyrus (frontal lobe), respectively. It should be noted that multiple ROI nodes from the Power atlas may correspond to the same anatomical region. For instance, Superior Frontal Gyrus appears three times (nodes 98, 99, and 102). This is because the Power atlas is a functional parcellation where distinct functional nodes can reside within the same macroscopic anatomical structure, each capturing different functional connectivity patterns. The distribution of these ROIs in the brain is shown in Figure 7. Detailed anatomical and functional network assignments for these regions are provided in Supplementary Table S2.

The results show that the top-ranked limbic lobe, frontal lobe, and thalamus exhibit strong correlations with MDD. The limbic lobe primarily consists of the cingulate gyrus, parahippocampal gyrus, and orbitofrontal cortex. Its main functions involve governing emotional processing, memory formation, and instinctive behaviors (such as fear responses). The frontal lobe’s key component is the prefrontal cortex, which governs higher cognition and emotional regulation. This is consistent with the findings of Huang et al. [39], further validating the effectiveness of the identified regions. Additionally, the thalamus ranked among the top model-attributed regions, likely due to its role in neural signal integration. This finding is consistent with Zhou et al. [40].

Spatial distribution of the top-10 model-attributed ROIs selected by SAGPooling, displayed from five standard perspectives: (a) left lateral, (b) right lateral, (c) medial, (d) ventral, and (e) dorsal views. Node labels indicate anatomical abbreviation and hemisphere (L, left; R, right). Node color reflects functional network assignment (red: default mode network, cyan: sensory/somatomotor hand, yellow: subcortical, blue: uncertain). Node size reflects selection frequency.

The identified model-attributed regions, including ACC and parahippocampal gyrus, are consistent with brain regions implicated in MDD pathophysiology by previous rs-fMRI studies. These regions are identified based on their selection frequency by the SAGPooling attention mechanism and should be considered model-attributed regions rather than independently validated biomarkers.

### 4.7. Limitations and Future Directions

Despite promising results, several limitations should be acknowledged. First, the study is limited by its single-site design, which restricts external validity and cross-site generalizability. This study deliberately employs single-site data to minimize cross-site confounding from scanner platforms, acquisition protocols, and recruitment demographics, thereby enabling a controlled evaluation of the core algorithmic performance. However, this design may introduce optimistic bias and limit generalizability. Future work will leverage the modular phenotypic-path architecture of heterogeneous graphs to encode cross-site factors—such as scanner platform, acquisition protocol, and recruitment site—as explicit edges (e.g., S–Site–S), enabling adaptive cross-site alignment through message passing. This approach treats site effects as learnable graph structures rather than nuisance variables to be removed.

Second, the publicly released REST-meta-MDD data lack subject-level motion parameters, quality-control metrics, and detailed clinical phenotyping (diagnostic instruments, medication status, symptom severity), precluding post hoc motion-confound analyses and subgroup stratification. The educational disparity between groups also represents a potential source of confounding. Future studies should utilize datasets with richer clinical phenotyping to enable more comprehensive subgroup and fairness analyses.

Third, the absolute Pearson correlation focuses on connectivity strength while discarding the directionality of functional connections, and the identified brain regions should be considered model-attributed regions rather than independently validated biomarkers. Future work should further explore signed connectivity, thresholded graphs, regularized precision matrices, and complementary attribution methods such as feature occlusion or integrated gradients to provide additional insights.

Fourth, a single 10-fold stratified run with a fixed random seed was employed to ensure experimental reproducibility. To further consolidate the statistical robustness of the findings, future work should conduct multiple independent repetitions with different random seeds, perform learning curve analyses with varying training set sizes, implement comprehensive hyperparameter scans, and extend the ablation study to include additional controlled baselines. Formal paired *t*-tests and multiplicity-corrected ablation experiments are also worth investigating.

Finally, the computational efficiency of the framework for practical clinical deployment warrants further investigation. The total training time of approximately 15 h on a single RTX 4090 GPU provides a practical reference. Future work will conduct a more comprehensive runtime and memory comparison against suitable baselines on identical hardware to better assess its potential for real-world applications. Additionally, calibration analysis, including calibration plots and Brier score, will be further explored to support potential clinical utility.

## 5. Conclusions

This study presents a novel HDGNN-Mamba2 framework aimed at differentiating participants with MDD from healthy controls. Spatiotemporal features derived from fMRI time series data are critical for MDD classification. To attain robust predictive performance through an end-to-end paradigm, the network constructs a heterogeneous graph by fusing multimodal datasets, encompassing these features alongside demographic and phenotypic information of individual subjects. Moreover, this approach is capable of identifying significant model-attributed regions relevant to disease classification.

First, a hybrid Mamba2-GNN module with a cross-attention fusion mechanism is designed, integrating Mamba with GNNs to capture individual spatiotemporal contextual features from fMRI data and to identify model-attributed regions. The Mamba module’s ability to efficiently handle long-range dependencies in sequential data, combined with a selective state space mechanism for encoding input sequences and learning representations, enables the capture of temporal dependencies in fMRI time series. Meanwhile, the Mamba2-GNN module employs graph attention networks to capture spatial relationships across brain regions. This enables the extraction of spatiotemporal features of individual subjects’ brain regions. Second, a heterogeneous global graph with a dynamic edge updating mechanism is constructed to learn inter-subject relationships, where embedding vectors are generated from individual brain features and non-imaging phenotypic information, collectively forming multimodal data. By aggregating information from different types of edges via a self-attention mechanism, an integrated heterogeneous global graph network embedding representation is obtained, incorporating multiple feature categories. Due to the limited sample size of the single-center dataset, the supervised contrastive learning module offers a modest improvement in classification performance, serving as an auxiliary enhancement to the primary classification loss. Finally, the HDGNN-Mamba2 captures the most essential embedded features by integrating multimodal data (including individual brain network features and global inter-subject association features), thereby achieving competitive classification performance for MDD. The testing results show that HDGNN-Mamba2 achieved an average classification accuracy of 83.88% after conducting ten-fold cross-validation. Through examining nodes with substantial contribution in the graph pooling layer, we additionally identified critical brain regions that exhibit discriminative patterns distinguishing participants with MDD from healthy controls. Furthermore, the HDGNN-Mamba2 can be adapted to other neuroimaging research areas. These benefits promote the computational classification of diverse neurological conditions and deepen the understanding of pathogenic mechanisms underlying complex neurological disorders. These results suggest that HDGNN-Mamba2 holds promise as a competitive algorithmic baseline for MDD classification research. While the framework demonstrates discriminative performance and identifies neurobiologically plausible model-attributed regions, it does not constitute a clinically validated diagnostic tool. Future work will incorporate probability calibration analysis and external validation on independent multi-site datasets to advance toward clinical utility.

## Figures and Tables

**Table 1:** Demographic characteristics of participants.

Characteristic	MDD	HC
Age (years)	38.74 ± 13.65	39.64 ± 15.87
Education (years)	10.78 ± 3.61	12.97 ± 3.94
Sex (Male/Female)	99/183	87/164

**Table 2:** Comparison results with baselines.

Method	ACC	Precision	Recall	F1
LR (demo)	0.5279 ± 0.0594	0.5471 ± 0.0639	0.5307 ± 0.0797	0.5392 ± 0.0846
SVM (demo)	0.5551 ± 0.0733	0.5229 ± 0.0630	0.6820 ± 0.0862	0.5905 ± 0.0664
MLP (img)	0.6417 ± 0.0543	0.6612 ± 0.0569	0.6759 ± 0.0670	0.6637 ± 0.0526
SVM (img + demo)	0.6550 ± 0.0570	0.7002 ± 0.0926	0.6558 ± 0.0789	0.6654 ± 0.0528
GCN (img + demo)	0.6918 ± 0.0387	0.7213 ± 0.0416	0.6024 ± 0.0483	0.6168 ± 0.0452
GAT (img + demo)	0.7053 ± 0.0414	0.6017 ± 0.0453	0.7162 ± 0.0428	0.5908 ± 0.0469
BrainGNN [37] (img + demo)	0.8112 ± 0.0282	0.8158 ± 0.0314	0.7387 ± 0.0347	0.7953 ± 0.0293
DeepGCN [38] (img + demo)	0.8264 ± 0.0318	0.8083 ± 0.0339	0.7208 ± 0.0380	0.8292 ± 0.0296
MFGCN [22] (img + demo)	0.8358 ± 0.0273	0.8452 ± 0.0287	0.8273 ± 0.0312	0.8528 ± 0.0263
HDGNN-Mamba2	0.8388 ± 0.0542	0.8397 ± 0.0438	0.8652 ± 0.0701	0.8490 ± 0.0551

Note: All baseline methods were evaluated on the same S20 cohort using identical ten-fold stratified cross-validation splits and the same preprocessing pipeline. LR (demo) and SVM (demo) employed demographic features as the sole input. MLP (img) took imaging features as the sole input. SVM (img + demo) concatenated imaging features with demographic variables. Methods with the (img + demo) suffix incorporated both imaging data and demographic variables. LR and SVM were implemented using Scikit-learn with default hyperparameters. GCN and GAT were implemented with two graph convolutional layers, a hidden dimension of 64, ReLU activation, dropout of 0.5, and the Adam optimizer with a learning rate of 0.001. The adjacency matrix for GCN and GAT was constructed using Pearson correlation, identical to the proposed method. BrainGNN, DeepGCN, and MFGCN were re-implemented following their original architectures with hyperparameters adopted from their respective publications. For all (img + demo) methods, demographic variables were included as one-hot encoded covariates.

**Table 3:** Ablation Experiments.

Ablation Settings	ACC	SEN	SPE	AUROC	AUPRC
HDGNN-Mamba2	0.8388 ± 0.0542	0.8652 ± 0.0701	0.8085 ± 0.0780	0.8819 ± 0.0565	0.8886 ± 0.0622
Conv-SSD + HGNN	0.7705 ± 0.0394	0.8033 ± 0.0572	0.7523 ± 0.0487	0.8090 ± 0.0234	0.8067 ± 0.0629
Conv-SSD	0.7363 ± 0.0739	0.7686 ± 0.0464	0.7244 ± 0.0583	0.7741 ± 0.0514	0.7712 ± 0.0366
RGNN + HGNN	0.7279 ± 0.0171	0.7604 ± 0.0389	0.7318 ± 0.0298	0.7654 ± 0.0331	0.7625 ± 0.0644
HGNN	0.5629 ± 0.0260	0.5908 ± 0.0173	0.5482 ± 0.0410	0.5936 ± 0.0375	0.5897 ± 0.0577
Mamba2-GNN	0.8033 ± 0.0536	0.8288 ± 0.0351	0.8087 ± 0.0587	0.8446 ± 0.0454	0.8422 ± 0.0426
without cross-attention	0.7871 ± 0.0304	0.8115 ± 0.0573	0.7733 ± 0.0188	0.8269 ± 0.0581	0.8248 ± 0.0325
without DEU	0.7953 ± 0.0613	0.8202 ± 0.0407	0.8020 ± 0.0580	0.8360 ± 0.0363	0.8421 ± 0.0561
without supervised contrastive learning	0.8150 ± 0.0476	0.8403 ± 0.0498	0.8037 ± 0.0471	0.8565 ± 0.0169	0.8631 ± 0.0464

**Table 4:** Experimental results based on different combinations of demographic data.

Demographic Data	Test-Acc	Precision	Recall	F1
Sex	0.8092 ± 0.0252	0.8135 ± 0.0375	0.8320 ± 0.0197	0.8220 ± 0.0451
Sex + Age	0.8280 ± 0.0284	0.8319 ± 0.0321	0.8508 ± 0.0243	0.8363 ± 0.0392
Sex + Education	0.8136 ± 0.0317	0.8212 ± 0.0289	0.8415 ± 0.0358	0.8310 ± 0.0296
Sex + Education + Age	0.8388 ± 0.0542	0.8397 ± 0.0438	0.8652 ± 0.0701	0.8490 ± 0.0551

## Data Availability

The REST-meta-MDD dataset is publicly available at http://rfmri.org/REST-meta-MDD (accessed on 12 August 2026). The code for HDGNN-Mamba2 is available from the corresponding author upon reasonable request.

## Supplementary Materials

The following supporting information can be downloaded at: https://www.mdpi.com/article/10.3390/brainsci16080872/s1, Table S1: Per-fold performance metrics for HDGNN-Mamba2; Table S2: Power atlas node information for the top 10 model-attributed regions.

## Abbreviations

The following abbreviations are used in this manuscript:

MDD	Major Depressive Disorder
fMRI	functional Magnetic Resonance Imaging
rs-fMRI	resting-state functional Magnetic Resonance Imaging
ROI	Region of Interest
FC	Functional Connectivity
GNN	Graph Neural Network
SSM	State Space Model
SSD	Structured State Space Duality
DEU	Dynamic Edge Updating
