# Supplementary material for: HDGNN-Mamba2: Mamba-Based Spatiotemporal Heterogeneous Dynamic Graph Neural Network for Major Depressive Disorder Classification
Source: Brain Sci. 2026 Aug 17;16(8):872. doi: 10.3390/brainsci16080872 (PMC13510404; doi:10.3390/brainsci16080872)
Supplement: Supplementary file 1 [file brainsci-16-00872-s001.zip › brainsci-4462387-supplementary.pdf]

Table S1. Per-fold performance metrics for HDGNN-Mamba2

Fold	N	TP	FP	TN	FN	ACC	SEN	SPE	Precision	F1	AUROC	AUPRC
1	54	21	5	20	8	0.7593	0.7241	0.8	0.8077	0.7636	0.7752	0.753
2	54	27	7	18	2	0.8333	0.931	0.72	0.7941	0.8571	0.9062	0.9109
3	54	23	3	23	5	0.8519	0.8214	0.8846	0.8846	0.8519	0.8777	0.8835
4	53	22	5	20	6	0.7925	0.7857	0.8	0.8148	0.8	0.8543	0.878
5	53	26	3	22	2	0.9057	0.9286	0.88	0.8966	0.9123	0.9371	0.9377
6	53	20	4	21	8	0.7736	0.7143	0.84	0.8333	0.7692	0.8114	0.8314
7	53	26	1	24	2	0.9434	0.9286	0.96	0.963	0.9455	0.9543	0.9634
8	53	26	7	18	2	0.8302	0.9286	0.72	0.7879	0.8525	0.8457	0.8423
9	53	26	7	18	2	0.8302	0.9286	0.72	0.7879	0.8525	0.9386	0.9556
10	53	27	6	19	1	0.8679	0.9643	0.76	0.8182	0.8852	0.9186	0.9306
Mean	—	24.4	4.8	20.3	3.8	0.8388	0.8652	0.8085	0.8397	0.8490	0.8819	0.8886
SD	—	2.5	2.0	2.3	2.5	0.0542	0.0701	0.0780	0.0438	0.0551	0.0565	0.0622

Table S2. Power atlas node information for the top 10 model-attributed regions

Rank	Power ROI	MNI (x, y, z)	Hemisphere	Anatomical Label	AAL Label	Brodmann Area	Functional Network
1	77	－13, －40, 1	Left	Parahippocampal Gyrus	Precuneus_L	—	Default mode
2	98	－10, 39, 52	Left	Superior Frontal Gyrus	Frontal_Sup_Medial_L	—	Default mode
3	113	－3, 42, 16	Left	Anterior Cingulate	Cingulum_Ant_L	BA 32	Default mode
4	90	－11, －56, 16	Left	Posterior Cingulate	Precuneus_L	—	Default mode
5	14	－14, －18, 40	Left	Cingulate Gyrus	—	BA 24	Sensory/somatomotor Hand
6	107	－7, 51, -1	Left	Medial Frontal Gyrus	Cingulum_Ant_L	—	Default mode
7	132	－31, 19, －19	Left	Inferior Frontal Gyrus	Frontal_Inf_Orb_L	—	Uncertain
8	234	9, －4, 6	Right	Thalamus	—	—	Subcortical
9	99	－16,29, 53	Left	Superior Frontal Gyrus	Frontal_Sup_L	BA 8	Default mode
10	102	13, 55,38	Right	Superior Frontal Gyrus	Frontal_Sup_Medial_R	BA 9	Default mode
